# Thematic Issue on Biofilms: Microbial Works of Art

**DOI:** 10.1111/1751-7915.12856

**Published:** 2017-09-22

**Table of Contents**

The contribution of microbial biotechnology to sustainable development goals

*K. Timmis, W. M. de Vos, J. L. Ramos, S. E. Vlaeminck, A. Prieto, A. Danchin, W. Verstraete, V. de Lorenzo, S. Y. Lee, H. Brüssow, J. K. Timmis and B. K. Singh* 984

Microbial technology with major potentials for the urgent environmental needs of the next decades

*W. Verstraete and J. de Vrieze* 988

Seven microbial bio‐processes to help the planet

*V. de Lorenzo* 995

**SDG 2: End hunger, achieve food security and improved nutrition and promote sustainable agriculture**

Tiny microbes, big yields: enhancing food crop production with biological solutions

*P. Trivedi, P. M. Schenks*,* M. D. Wallenstein and B. K. Singh* 999

Increased nutritional value in food crops

*N. Goicoechea and M. C. Antolín* 1004

Beneficial microbial signals from alternative feed ingredients: a way to improve sustainability of broiler production?

*F. Van Immerseel, V. Eeckhaut, R. J. Moore, M. Choct and R. Ducatelle* 1008

Biofloc technology application in aquaculture to support sustainable development goals

*P. Bossier and J. Ekasari* 1012

Microalgae, old sustainable food and fashion nutraceuticals

*J. L. Garcia, M. de Vicente and B. Galán* 1017

Hunger and microbiology: Is a low gastric acid‐induced bacterial overgrowth in the small intestine a contributor to malnutrition in developing countries?

*S. A. Sarker, T. Ahmed and H. Brüssow* 1025

**Goal 3. Ensure healthy lives and promote well‐being for all at all ages**

Microbially derived biosensors for diagnosis, monitoring and epidemiology

*H.‐J. Chang, P. L. Voyvodic, A. Zúñiga and J. Bonnet* 1031

Roads to advanced vaccines: influenza case study

*P. Riese and C. A. Guzmán* 1036

Infection therapy: the problem of drug resistance – and possible solutions

*H. Brüssow* 1041

Microbial approaches for targeting antibiotic‐resistant bacteria

*W. F. Wong and M. Santiago* 1047

Strategies for combating persister cell and biofilm infections

*T. K. Wood* 1054

Sustainable therapies by engineered bacteria

*B. Álvarez and L. Á. Fernández* 1057

Metal‐based antimicrobial strategies

*R. J. Turner* 1062

The contribution of microbial biotechnology to sustainable development goals: microbiome therapies

*P. W. O'Toole and M. Paoli* 1066

Synbiotic approaches to human health and well‐being

*T. Gurry* 1070

Tumor‐targeting bacteria‐based cancer therapies for increased specificity and improved outcome

*S. Felgner, V. Pawar, D. Kocijancic, M. Erhardt and S. Weiss* 1074

Hygiene: microbial strategies to reduce pathogens and drug resistance in clinical settings

*E. Caselli* 1079

The DIY Digital Medical Centre

*J. K. Timmis and K. Timmis* 1084

**Goal 6. Ensure availability and sustainable management of water and sanitation for all**

Microbial biotechnologies for potable water production

*S. J. Fowler and B. F. Smets* 1094

Current and future microbiological strategies to remove As and Cd from drinking water

*J. M. Byrne and A. Kappler* 1098

Microbial biotechnology and circular economy in wastewater treatment

*P. H. Nielsen* 1102

Controlling cyanobacterial harmful blooms in freshwater ecosystems

*H. W. Paerl* 1106

**Goal 7. Ensure access to affordable, reliable, sustainable and modern energy for all**

Green biofuels and bioproducts: bases for sustainability analysis

*J. L. Ramos, F. García‐Lorente, M. Valdivia and E. Duque* 1111

Bioelectricity (electromicrobiology) and sustainability

*K. H. Nealson* 1114

Advances and bottlenecks in microbial hydrogen production

*A J. Stephen, S. A. Archer, R. L. Orozco and L. E. Macaskie* 1120

Biogas

*C. M. Plugge* 1128

Fungal nanoscale metal carbonates and production of electrochemical materials

*Q. Li and G. M. Gadd* 1131

**Goal 8. Promote sustained, inclusive and sustainable economic growth, full and productive employment and decent work for all**

The contribution of microbial biotechnology to economic growth and employment creation

*K. Timmis, V. de Lorenzo, W. Verstraete, J. L. Ramos, A. Danchin, H. Brüssow, B. K. Singh and J. K. Timmis* 1137

**Goal 11. Make cities and human settlements inclusive, safe, resilient and sustainable**

Microbial biotechnology approaches to mitigating the deterioration of construction and heritage materials

*P. Junier and E. Joseph* 1145

Microbial communities as biosensors for monitoring urban environments

*F. Ling* 1149

Bioprotection of the built environment and cultural heritage

*G. M. Gadd and T. D. Dyer* 1152

Architects of nature: growing buildings with bacterial biofilms

*M. Dade‐Robertson, A. Keren‐Paz, M. Zhang and I. Kolodkin‐Gal* 1157

**Goal 12. Ensure sustainable consumption and production patterns**

The future of biologically inspired next‐generation factories for chemicals

*C. Schmidt‐Dannert* 1164

Gas fermentation for commodity chemicals and fuels

*F. R. Bengelsdorf and P. Dürre* 1167

Metallic bionanocatalysts: potential applications as green catalysts and energy materials

*L. E. Macaskie, I. P. Mikheenko, J. B. Omajai, A. J.Stephen, and J. Wood* 1171

Bacterial cellulose as an example product for sustainable production and consumption

*W. D. Jang, J. H. Hwang, H. U. Kim, J. Y. Ryu and S. Y. Lee* 1181

Microbial melanins for radioprotection and bioremediation

*R. J. B. Cordero, R. Vij and A. Casadevall* 1186

Biomining of metals: how to access and exploit natural resource sustainably

*C. A. Jerez* 1191

Recovery of precious metals from waste streams

*J. He and A. Kappler* 1194

Metal and metalloid biorecovery using fungi

*X. Liang and G. M. Gadd* 1199

How to access and exploit natural resources sustainably: petroleum biotechnology

*A. Sherry, L. Andrade, A. Velenturf, B. Christgen, N. D Gray and I. M. Head* 1206

The contribution of microbially produced nanoparticles to sustainable development goals

*M. E. Cueva and L. E. Horsfall* 1212

**Goal 13. Take urgent action to combat climate change and its impacts**

About how to capture and exploit the CO_2_ surplus that nature, per se, is not capable of fixing

*M. S. Godoy, B. Mongili, D. Fino and M. A. Prieto* 1216

Harnessing microbiome‐based biotechnologies for sustainable mitigation of nitrous oxide emissions

*H.‐W. Hu, J.‐Z. He and B. K. Singh* 1226

**Goal 14. Conserve and sustainably use the oceans, seas and marine resources for sustainable development**

Microbial biotechnology addressing the plastic waste disaster

*T. Narancic and K. E. O'Connor* 1232

The contribution of microbial biotechnology to mitigating coral reef degradation

*K. Damjanovic, L. L. Blackall, N. S. Webster and M. J. H. van Oppen* 1236

**Goal 15. Protect, restore and promote sustainable use of terrestrial ecosystems, sustainably manage forests, combat desertification, and halt and reverse land degradation and halt biodiversity loss**

Soil and brownfield bioremediation

*M. Megharaj and R. Naidu *1244

Microbial biotechnology as a tool to restore degraded drylands

*F. T. Maestre, R. Solé and B. K. Singh* 1250

**Enabling Technologies of microbial approaches towards attainment of sustainable development goals**

Systems metabolic engineering as an enabling technology in accomplishing sustainable development goals

*D. Yang, J. S. Cho, K. R. Choi, H. U. Kim and S. Y. Lee* 1254

The contribution of bacterial genome engineering to sustainable development

*D. R. Reuß, F. M. Commichau and J. Stülke* 1259

Synthetic microbiology: from analogy to methodology

*V. de Lorenzo* 1264

Bioprocess scale‐up/down as integrative enabling technology: from fluid mechanics to systems biology and beyond

*F. Delvigne, R. Takors, R. Mudde, W. van Gulik and H. Noorman* 1267

